# Extracellular vesicles mediate immune regulation in acute kidney injury

**DOI:** 10.3389/fimmu.2026.1952566

**Published:** 2026-09-01

**Authors:** Yu-Qi Fu, Tao-Tao Tang, Si-Jie Chen, Bin Wang, Bi-Cheng Liu

**Affiliations:** Institute of Nephrology, Zhongda Hospital, Jiangsu Provincial Key Laboratory of Renal Injury and Repair, Southeast University School of Medicine, Nanjing, Jiangsu, China

**Keywords:** acute kidney injury, exosomes, extracellular vesicles, renal immune regulation, single-vesicle analysis

## Abstract

Acute kidney injury (AKI) arises from diverse insults that trigger distinct immune responses, and an integrative framework for intercellular communication is now emerging. Extracellular vesicles (EVs), membrane-enclosed particles carrying proteins, microRNAs, lipids, and metabolites, mediate crosstalk between renal parenchymal cells and immune effectors to shape inflammation and repair. This review examines three dimensions of EV biology in AKI. This review examines three dimensions of EV biology in AKI, applying the term ‘EV’ throughout in accordance with MISEV2023 guidance unless the cited primary study has experimentally established subtype origin. First, we outline the molecular basis of EV-mediated immune signaling by contrasting vesicular communication with soluble cytokine and cell-contact pathways, highlighting cargo stability, tissue tropism, and multi-signal integration as distinguishing features. Second, we describe how EV composition shifts after injury: tubular epithelial cells, podocytes, and endothelial vesicles become enriched in damage-associated patterns and pro-inflammatory microRNAs, whereas immune cell-derived EVs propagate or resolve inflammation depending on polarization and disease phase. Third, we compare vesicular signaling across four AKI etiologies (allograft rejection, sepsis, nephrotoxicity, and ischemia-reperfusion), noting that EV cargo, cellular origin, and immune targets differ markedly by insult type and evolve through early, peak, and reparative phases. Advances in single-vesicle proteomics, intravital imaging, and kidney organoid systems now enable functional dissection of EV heterogeneity at unprecedented resolution. Key gaps remain: the limited mechanistic definition of dendritic cell- and T cell-derived EVs in non-transplant settings, the underdeveloped vesicular framework for renal ischemia-reperfusion, and the absence of standardized clinical assays. Addressing these limitations is essential for translating mechanistic insights into etiology-stratified diagnostics and stage-matched immunomodulatory interventions.

## 1 Introduction

Extracellular vesicles (EVs) are now recognized as fundamental mediators of intercellular communication under both physiological and pathological conditions (1–3). Given its dense vascularization and complex immune microenvironment, the kidney relies heavily on EV-based signaling to maintain homeostasis and respond to injury. By transporting biologically active cargo, including proteins, microRNAs, lipids, and metabolites, EVs modulate cellular behavior at both local and systemic levels (4, 5). Acute kidney injury (AKI) is a common, life-threatening clinical syndrome in which disease initiation, progression, and outcomes are tightly coupled to the dynamic dysregulation of renal immune homeostasis (6, 7). Accumulating evidence indicates that EVs are not merely passive byproducts of renal injury but actively regulate communication between innate and adaptive immunity, thereby shaping the balance between inflammation and tissue repair (8–10). Recent reviews have comprehensively examined EVs as both diagnostic tools and therapeutic targets across multiple organ systems (11, 12), and their fundamental role in kidney disease is now well established (13). In the context of AKI, EVs have been implicated in biomarker discovery, therapeutic intervention, and immune regulation (14).

This review examines how EVs function as immunological messengers in AKI, focusing on the mechanisms by which they program resident dendritic cells and macrophages, initiate innate immune activation, and mediate crosstalk between resident renal cells and infiltrating immune cells across distinct injury stages. By integrating recent advances in single-vesicle analytical technologies, we aim to clarify EV-centered immunopathological mechanisms in AKI and identify promising directions for future diagnostic and therapeutic development.

## 2 EV biology relevant to immune regulation

According to the Minimal Information for Studies of Extracellular Vesicles (MISEV) guidelines, released by the International Society for Extracellular Vesicles (ISEV) in 2018 and updated in 2023, EVs are commonly classified into three subtypes based on biogenesis: exosomes (30–150 nm; derived from multivesicular bodies), microvesicles (100–1000 nm; formed by outward budding of the plasma membrane), and apoptotic bodies (500–5000 nm; released during programmed cell death) (15, 16). In accordance with MISEV2023 recommendations (16), the present review uses the generic term ‘EV’ to refer to membrane-enclosed particles characterized by size, surface markers, and cargo. The terms ‘exosome,’ ‘microvesicle,’ and ‘apoptotic body’ are reserved for studies in which biogenesis has been experimentally demonstrated; original nomenclature is preserved in parentheses at first mention per cited primary report. However, increasing evidence suggests that classification based solely on size and origin does not adequately capture EV functional heterogeneity. Importantly, flow cytometry experiments on EVs now require adherence to minimum reporting standards to ensure reproducibility (17), reflecting the field’s increasing methodological rigor. A single cell type may release EV subpopulations with distinct or even opposing immunological effects, whereas EVs from different cellular sources may share highly similar immunoregulatory cargo (18). Consequently, the field is shifting from morphology-based to function-oriented classification, emphasizing that EV activity is determined by the integrated effects of cargo composition, surface molecular signatures, and the recipient cell microenvironment (2, 19). Recent work has identified additional EV populations released specifically in the context of cell death. Apoptotic exosome-like vesicles (AEVs) constitute a distinct category that differs from classical apoptotic bodies in both size (~100 nm) and biogenesis: their formation depends on caspase-3 activation and autolysosomes rather than on membrane blebbing alone, and they carry a cargo enriched in DAMPs and immunogenic signals capable of activating innate immune receptors in recipient cells (20–22). Unlike classical apoptotic bodies, which are rapidly cleared by phagocytosis and are generally immunologically silent, AEVs appear to sustain and amplify sterile inflammatory signaling following cell death. Although the role of AEVs in AKI has not yet been directly examined, their DAMP-enriched cargo and the well-established occurrence of regulated cell death in the injured tubule make them candidates for further investigation as mediators of early immune activation in this setting.

Compared with soluble cytokine signaling and direct cell–cell contact, EV-mediated communication exhibits several distinctive properties well suited to the anatomical complexity of the kidney. Soluble cytokines undergo rapid dilution and degradation, limiting their range, while cell-contact signaling requires precise spatiotemporal co-localization (23). EVs circumvent both constraints. The lipid bilayer protects vesicular cargo from extracellular nucleases and proteases, allowing urinary EV-associated mRNA to remain stable at room temperature for hours, whereas free mRNA degrades within minutes (24). A recent multi-day study has confirmed the robustness of urinary EV-derived miRNA profiles over time, although urine concentration can impact absolute quantification (25). Surface integrins confer directional targeting. This principle was first demonstrated experimentally in tumor-derived exosomes: Hoshino et al. showed that exosomes expressing α6β4 and α6β1 home preferentially to lung tissue, whereas αvβ5-positive counterparts accumulate in the liver (26). This mechanism has since been examined in the kidney. Shen et al. systematically reviewed the integrin–exosome axis in kidney disease, summarizing evidence that integrins are expressed on renal parenchymal cell-derived exosomes, participate in exosome biogenesis and cargo sorting, and mediate exosome uptake by recipient renal and immune cells, including in the context of tubular injury and AKI (27).

Cargo relevant to renal immunity falls into four categories. Damage-associated molecular patterns (DAMPs), such as HMGB1, mitochondrial DNA, and heat-shock proteins, engage innate receptors and initiate inflammatory cascades (28). Regulatory microRNAs, including miR-155, miR-146a, and miR-21, modulate immune cell differentiation and effector function post-transcriptionally (29, 30). Metabolism-related cargo, such as arginase-1, can reshape local substrate availability and influence T-cell function (31, 32). Surface proteins, including MHC class I/II, CD80/CD86, PD-L1, ICAM-1, integrins, and complement regulators (CD55, CD59), determine target-cell recognition (33, 34). Furthermore, individual EVs can co-deliver peptide-MHC complexes, costimulatory molecules, and regulatory microRNAs (35), producing synchronized multisignal transmission whose efficiency exceeds the sum of individual molecular signals (36, 37). A fifth category, proteasome-associated cargo, is gaining recognition as an immunologically relevant component of the EV payload. 20S proteasome subunits have been identified within EVs, and vesicle-delivered proteasomes retain catalytic activity in recipient cells, as demonstrated in a recent study showing that EV-mediated 20S proteasome transfer enhances proteolytic substrate clearance in target cells (38). Although direct AKI-specific evidence is currently limited, we propose this category as a hypothesis-generating addition to the renal-EV cargo framework. Separately, engineered EVs have been shown to co-deliver immunomodulatory cargo, such as IL-10 or miR-125b-5p, achieving therapeutic efficacy in ischemic AKI that exceeds equivalent free-drug dosing (39, 40). Together, these features provide a mechanistic rationale for EV involvement in renal immune events. Although the kidney lacks organized lymphoid structures in its uninjured state, it is densely perfused and populated by resident immune cells. It should be noted, however, that tertiary lymphoid structures (TLS)—organized ectopic lymphoid aggregates—can develop within the renal interstitium during chronic inflammation or sustained injury (41), TLS contribute to local antigen presentation and adaptive immune regulation, and, by analogy with other inflamed tissues, may themselves generate EVs that shape the renal immune milieu. Whether TLS-derived EVs participate in AKI pathophysiology or in the transition to chronic kidney disease remains to be investigated and represents a potentially important research direction. Classical dendritic-cell migration from peripheral tissues to draining lymph nodes typically requires days (42), a timeframe that cannot readily explain the rapid adaptive immune activation observed within hours of AKI onset. As a working hypothesis, EVs may bypass this kinetic constraint by disseminating immune-relevant cargo from injured tissue to distant immune compartments via the circulation within minutes to hours; however, this model has not been directly tested in AKI, and defining the corresponding time course, anatomical routes, and cargo identity *in vivo* will require dedicated tracer studies. Concurrently, EVs may support local signal exchange between innate and adaptive immune cells directly within renal tissue, reducing reliance on the lymph node-dependent pathway of classical antigen presentation (43). In a richly vascularized but lymphoid-poor organ such as the kidney, this EV-mediated route offers a mechanistically plausible but currently unvalidated basis for rapid, localized immune regulation during AKI, and motivates the central question of this review: how, when, and from which cellular sources do EVs contribute to the immune trajectory of the injured kidney (44, 45)?

## 3 Evidence map: EV sources, cargo, and recipient immune cells in AKI

### 3.1 The renal immune microenvironment as a context for EV signaling

The kidney receives approximately 20–25% of resting cardiac output and filters nearly 180 L of glomerular filtrate (primary urine) daily (46), continuously exposing it to circulating immune complexes, complement proteins, microbial products, and metabolic byproducts (47). Resident renal cells, including proximal and distal tubular epithelial cells, podocytes, mesangial cells, glomerular endothelial cells, peritubular endothelial cells, and pericytes, are active participants in immune regulation rather than passive targets of injury. Pericytes, which ensheath the peritubular capillaries and regulate microvascular tone, are increasingly recognized as contributors to interstitial inflammation and fibrosis (48). Glomerular and peritubular endothelial cells differ substantially in their gene expression profiles, susceptibility to injury signals, and responses to inflammatory stimuli during AKI (49). The peritubular capillary network—a distinct vascular compartment downstream of the glomerulus—is particularly susceptible to rarefaction during AKI and is increasingly recognized as a key determinant of the transition from AKI-to-CKD transition (50, 51). Given that endothelial cells lining the peritubular capillaries represent a major potential source of EVs during ischemic and inflammatory insults, this vascular compartment warrants attention as both a generator and a target of EV-mediated immune signals in AKI. TECs express pattern-recognition receptors (TLR2, TLR4, TLR9), inflammasome components, and complement receptors, enabling direct sensing of DAMPs (52, 53), whereas podocytes express MHC class II and B7 family molecules, conferring antigen-presenting capacity independent of hematopoietic origin (54). Furthermore, the kidney harbors abundant tissue-resident immune cell populations, including macrophages and dendritic cells, which collectively constitute the first line of renal immune surveillance (55, 56).

Traditional immune cell-centric models of AKI emphasize leukocyte infiltration and inflammatory cascades, such as M1/M2 macrophage polarization shifts and T-cell subset imbalance (57, 58), yet fail to explain several key observations. Recent mechanistic studies have elucidated major signaling pathways and key mediators governing macrophage function in AKI (59), including the PI3K-Akt-CCND2 axis that couples metabolic reprogramming with M1 polarization (60), and redox-dependent macrophage reprogramming dynamics in acute inflammation (61). A substantial proportion of patients with septic AKI develop irreversible functional impairment despite minimal renal immune cell infiltration (62, 63); T-cell-deficient mice still develop marked tubular injury following ischemia-reperfusion (64); and the classical antigen presentation pathway, which requires several days, cannot account for the rapid adaptive immune activation observed within 6–12 hours of AKI onset (65, 66). These discrepancies suggest the existence of immune regulatory mechanisms that precede or operate independently of overt immune cell recruitment. EVs may explain selected early communication events by enabling immune signal transmission before massive leukocyte infiltration, mediating long-range communication without direct cell contact, and integrating multiple regulatory cues within the renal microenvironment (5, 67), thereby addressing the limitations of conventional paradigms.

### 3.2 EVs from renal parenchymal cells

Renal parenchymal cells are a major source of EVs during AKI, and their vesicles exert profound effects on local and systemic immune responses across all phases of injury. A growing body of evidence documents the contributions of tubular epithelial cells, podocytes, and endothelial cells to the renal EV landscape, although the depth of mechanistic characterization differs substantially across these populations (68, 69).

#### 3.2.1 EVs from tubular epithelial cells

In the healthy kidney, TECs continuously secrete EVs containing aquaporin-2 (AQP2) and the sodium-chloride cotransporter (NCC), contributing to the paracrine regulation of tubular reabsorption (70). Under injurious conditions, the cargo profile of TEC-derived EVs shifts markedly toward a pro-inflammatory and pro-fibrogenic composition, though primary evidence for this shift derives from specific experimental models rather than from a unified injury paradigm.

##### 3.2.1.1 Immune activation cargo

In a rodent model of albumin-induced tubulointerstitial injury supported by matched *in vitro* TEC experiments, Lv et al. showed that TEC-derived EVs are enriched in the chemokine CCL2, which recruits and activates infiltrating macrophages to drive tubulointerstitial inflammation (71). The same group subsequently demonstrated that TEC exosomes deliver miR-19b-3p to macrophages and promote M1 polarization in a proteinuric kidney-injury model, providing direct evidence that stressed epithelial cells transmit inflammatory instructions to intrarenal phagocytes via vesicular microRNAs (4). Under hypoxic conditions, HIF-1α activation in TECs induces exosomal miR-23a, which is transferred to macrophages and amplifies tubulointerstitial inflammation through a second, oxygen-sensing route of TEC–macrophage vesicular crosstalk (72).

##### 3.2.1.2 Fibrogenic transition cargo

Borges et al. showed that TGF-β1-containing EVs released from injured TECs activate interstitial fibroblasts and initiate the shift from regenerative to fibrogenic remodeling, linking acute epithelial injury to sustained interstitial pathology (73). More recently, Zhong et al. demonstrated that senescent TEC-derived EVs transport miR-20a and miR-21 to macrophages, inducing macrophage-to-myofibroblast transition in the fibrotic phase following ischemia-reperfusion injury (74). These findings indicate that TEC-derived EVs function as stage-dependent mediators: pro-inflammatory in the acute phase and pro-fibrogenic during the transition to chronic injury.

#### 3.2.2 EVs from podocytes

Podocyte-derived EVs contain latent TGF-β1 and anti-inflammatory microRNAs that maintain glomerular immune tolerance by suppressing NF-κB signaling in resident macrophages under homeostatic conditions (75).

As terminally differentiated, non-regenerating cells, podocytes are particularly vulnerable to irreversible loss once regulated cell death programs — including apoptosis and the more recently characterized non-apoptotic pathways such as pyroptosis and ferroptosis — are engaged; EVs have been identified as a principal conduit through which death and survival signals are relayed to and from the podocyte compartment, positioning EV-mediated crosstalk as a determinant of whether podocyte injury resolves or progresses to irreversible loss (76). Alongside cell-death regulation, autophagy — a key protective mechanism against podocyte injury — is itself modulated by EV cargo, with several autophagy-regulating microRNAs and proteins shown to be selectively packaged into podocyte-derived and podocyte-targeting EVs (77). Podocytes generate not only conventional exosomes and microvesicles but also migrasomes — large, migration-associated vesicles with distinct biogenesis and cargo — suggesting a more differentiated intercellular signaling repertoire than previously appreciated (78). Under injurious conditions, podocytes release EVs carrying MHC class II molecules and B7 family costimulatory signals capable of activating local T cells within the glomerulus (54, 79).

Exogenous EVs also appear capable of protecting podocytes under stress: endothelial progenitor cell-derived EVs have been shown to confer protection to glomerular endothelial cells and podocytes in models of glomerular injury, hinting at a paracrine endothelial-podocyte protective axis that may be relevant during the microvascular stress accompanying AKI (80).

Taken together, these observations indicate that podocyte EV biology extends well beyond antigen presentation to encompass cell-death regulation, autophagic signaling, and heterotypic vesicle diversity. Nonetheless, most of this evidence derives from chronic glomerular injury models; establishing whether these EV-mediated programs operate similarly during AKI is an open question that warrants dedicated investigation.

#### 3.2.3 EVs from endothelial cells

Endothelial cell-derived EVs carry vasoactive molecules, growth factors, and microRNAs that collectively contribute to the maintenance of renal microvascular homeostasis and a local anti-inflammatory state under physiological conditions (81). In the context of renal injury, endothelial EVs take on additional functional roles: endothelial cell-targeted EVs have been shown to confer renoprotection against ischemic injury in experimental models (82), and endothelial EVs carrying miR-423-5p regulate peritubular microvascular homeostasis and renal function following ischemia-reperfusion, partly through modulation of endothelial-immune cell interactions (83). Endothelial progenitor cell-derived EVs have additionally been shown to promote angiogenesis and vascular repair, broadening the reparative potential of this EV population (84).

#### 3.2.4 EV-based biomarker evidence in renal parenchymal cell injury

Beyond mechanistic cargo studies, human urinary EV analyses have identified miR-29c as a biofluid marker that tracks tubulointerstitial fibrosis (85), and exosomal Fetuin-A as a urinary marker of tubular injury in both rodent cisplatin nephrotoxicity and human ICU AKI (86). Collectively, the biomarker data reviewed here complement the mechanistic cargo studies above and are incorporated in the evidence summary in **Table 1**.

**Table 1:** EV-mediated immune signaling across AKI etiologies and evidence tiers.

AKI etiology/context	EV source (cell of origin)	Representative cargo	Recipient cell/target	Immune/functional effect	Evidence tier	Evidence type	Key reference
Allograft rejection (perioperative phase)	Donor kidney tissue-derived EVs (mixed parenchymal)	Donor HLA molecules; mitochondrial fragments; tissue-specific transcripts	Recipient spleen/draining lymph node APCs	Alloantigen delivery preceding overt graft dysfunction	II — Human transplant biomarker study	Biomarker/Associative	Vallabhajosyula 2017 (107)
Allograft rejection (T-cell–mediated)	Kidney allograft EVs	miR-218-5p	Recipient CD4^+^ T cells	Th17/Treg skew toward pro-inflammatory phenotype	II — Human allograft with functional validation	Mechanistic	Rutman 2022 (108)
Allograft rejection (diagnostic signature)	Urinary exosomes	CXCL9, CXCL11, CD3, CD4, IFNG, PRF1 mRNA panel	— (biofluid readout)	Discriminates acute rejection from stable graft function and from BKVN	II — Human multi-center validated cohort	Biomarker/Associative	El Fekih 2021 (111)
Allograft rejection (detection platform)	Urinary transplant EVs	Rejection-associated protein/RNA cargo	— (biofluid readout)	Microfluidic non-invasive detection	III — Human proof-of-concept platform	Platform	Park 2017 (112)
Sepsis-AKI (early bacteremic phase)	Plasma exosomes (circulating)	Inflammatory cytokines; NF-κB- and cell-cycle-related microRNAs; reactive oxygen species generators	Renal microvasculature (inferred)	Systemic pro-inflammatory and redox signaling	III — Human plasma evidence; renal effects inferred	Biomarker/Associative	Reithmair 2017 (117); Real 2018 (118)
Sepsis-AKI (peak inflammation)	Septic macrophage–derived exosomes	SIRT1-suppressing cargo	Glomerular endothelial cells	Capillary rarefaction; impaired oxygen delivery	I — Rodent S-AKI + matched *in vitro* endothelial studies	Mechanistic	Zhang 2023 (89)
Sepsis-AKI (diagnostic signature)	Single urinary EVs	Complement receptor CD35 (CR1)	— (biofluid readout)	Distinguishes S-AKI from non-septic AKI and from sepsis without renal involvement	I — Validated human ICU cohort, single-vesicle proteomics	Biomarker/Associative	Li 2025 (124)
Sepsis-AKI (resolution/conditioning)	Plasma exosomes after remote ischemic preconditioning	miR-21	Renal tubular cells	Suppresses programmed cell death; attenuates S-AKI severity	I — Rodent S-AKI; awaits independent replication	Mechanistic	Pan 2019 (123)
Nephrotoxic AKI (early biomarker)	Urinary EVs (mixed renal origin)	Fetuin-A	— (biofluid readout)	Tracks tubular injury across rodent cisplatin model and human ICU AKI	I — Rat cisplatin model + ICU AKI patients	Biomarker/Associative	Zhou 2006 (86)
Nephrotoxic AKI (cisplatin mechanism)	TEC-derived exosome/TFEB-MVB axis	Pro-apoptotic and pro-inflammatory EV cargo (release suppressed)	Autocrine/paracrine on TEC	TFEB-mediated MVB regulation attenuates tubular apoptosis	I — Rodent cisplatin-AKI	Mechanistic	Xu 2026 (127)
Ischemia-reperfusion AKI (fibrogenic axis)	Injured TEC-derived exosomes	TGF-β1	Interstitial fibroblasts	Fibroblast activation; regenerative-to-fibrogenic transition	I — Rodent renal IRI	Mechanistic	Borges 2013 (73)
Ischemia-reperfusion AKI (innate injury)	Neutrophil-derived vesicles and NETs	Complement-linked chromatin–histone–protease complexes	TEC	Complement (C3aR)-dependent NET formation aggravates tubular injury	I — Rodent renal IRI (C3aR-KO model)	Mechanistic	Han 2023 (97)

This table summarizes current evidence for EV involvement in AKI immune regulation, organized by etiology, EV source, cargo, recipient cell, immune effect, evidence tier, and evidence type. Evidence type distinguishes mechanistic studies (direct experimental demonstration of a causal cargo–recipient–effect relationship in renal or transplant models) from biomarker/associative studies (EV cargo correlates with clinical or experimental outcome but causal specificity has not been established) and platform studies (detection capability demonstrated without paired mechanistic validation). Cross-organ analogy entries (Tier IV; hepatotoxic, pulmonary, and cardiocerebral ischemia models) have been moved to **Supplementary Table 1** to reduce table density; they are retained in that supplementary table to support hypothesis generation in nephrotoxic and IRI-AKI settings. Evidence-tier definitions: Tier I, direct *in vivo* AKI evidence (rodent or human) with functional or clinical validation; Tier II, kidney transplant or non-AKI kidney disease *in vivo* evidence; Tier III, *in vitro* renal cell/organoid evidence, or human biofluid evidence without paired renal functional validation. All reference numbers correspond to the consolidated reference list. APC, antigen-presenting cell; BKVN, BK virus nephropathy; C3aR, complement component 3a receptor; CR1, complement receptor 1 (CD35); EV, extracellular vesicle; HLA, human leukocyte antigen; ICU, intensive care unit; IRI, ischemia-reperfusion injury; KO, knockout; MVB, multivesicular body; NET, neutrophil extracellular trap; S-AKI, sepsis-associated acute kidney injury; TEC, tubular epithelial cell; TFEB, transcription factor EB.

### 3.3 EVs from immune cells in AKI

Beyond parenchymal sources, immune cell-derived EVs play essential roles in the initiation, amplification, and resolution of renal inflammation during AKI. Macrophages, dendritic cells, neutrophils, and lymphocytes all release EVs that influence renal immune homeostasis (87, 88). Macrophage-derived EVs exhibit polarization-dependent heterogeneity: vesicles from classically activated macrophages propagate inflammatory signaling to TECs and endothelial cells, aggravating microvascular dysfunction and contributing to injury propagation in sepsis-associated AKI (S-AKI) (89, 90). Recent work has further dissected mechanisms of EV-mediated macrophage regulation in renal inflammatory diseases, revealing that macrophage-derived EVs propagate inflammation in AKI (91). Mechanistically, maintenance of the M1 macrophage phenotype in acute renal inflammation depends on the pattern recognition receptor Mincle, indicating that EV-producing macrophage identity is shaped by innate pattern-recognition inputs in addition to classical polarization cues (92). Conversely, macrophage-derived microvesicles have been engineered as drug-delivery vehicles for kidney-targeted dexamethasone, achieving organ-selective anti-inflammatory efficacy and highlighting the therapeutic potential of exploiting endogenous EV tropism (93). In addition, macrophage-specific KLF4 expression attenuates TNFα-mediated kidney injury and fibrosis, indicating that the transcriptional state of EV-releasing macrophages critically shapes downstream renal inflammatory output (94). Mechanistic details of M1-macrophage-EV action are consolidated here rather than repeated in Section 4.2.

Neutrophils release EVs that are mobilized rapidly following inflammatory stimulation and carry a cargo of granule proteins, cytokines, and microRNAs distinct from the chromatin-histone-protease scaffold that defines neutrophil extracellular traps (NETs), a related but mechanistically separate neutrophil product discussed below. Direct evidence for neutrophil-derived EV release specifically within the injured kidney remains limited, and much of the current understanding is extrapolated from *in vitro* stimulation of isolated neutrophils with bacterial components or chemokines rather than from renal injury models per se (95). Nonetheless, the functional profile of this EV population appears to extend beyond a purely pro-inflammatory role: in a murine sepsis model, neutrophil-derived EVs carrying superoxide dismutase 2 were shown to restrain sepsis-associated coagulopathy (96), indicating that neutrophil EVs can mediate both injury and repair depending on their cargo and the context of release — a duality that invites further investigation in the setting of AKI.

NETs warrant separate consideration because, despite arising from the same cellular source, they are extracellular chromatin-protein structures extruded during NETosis rather than membrane-bound vesicles, and evidence concerning NET formation should not be conflated with evidence of EV-mediated signaling. Han et al. demonstrated that in renal ischemia-reperfusion injury, genetic deletion of the complement C3a receptor reduces both NET formation and tubular damage (97), establishing a complement-dependent mechanism for NET-driven injury; this finding constitutes direct *in vivo* evidence for the NET axis in renal IRI but does not itself speak to neutrophil-derived EV biology. The two processes are not entirely independent. Emerging evidence indicates that neutrophil-derived EV cargo can modulate NET architecture and stability (98), though this crosstalk has not yet been characterized in the kidney. We therefore treat neutrophil-derived EVs, NETs, and their potential interaction as three distinct lines of evidence in this review, considering them as related but mechanistically separable effector pathways at the interface of complement activation and innate immunity in AKI.

DC-derived EVs are well characterized in transplant settings, where donor DC-derived exosomes traffic to recipient lymphoid organs and prime alloreactive T cells (99–101). However, whether analogous DC-EV-mediated antigen presentation occurs in non-transplant AKI, or contributes to autoreactive or infection-driven T-cell responses, remains unclear. No study has directly demonstrated an effector role for DC-EVs in non-transplant renal injury.

**Figure 1:**
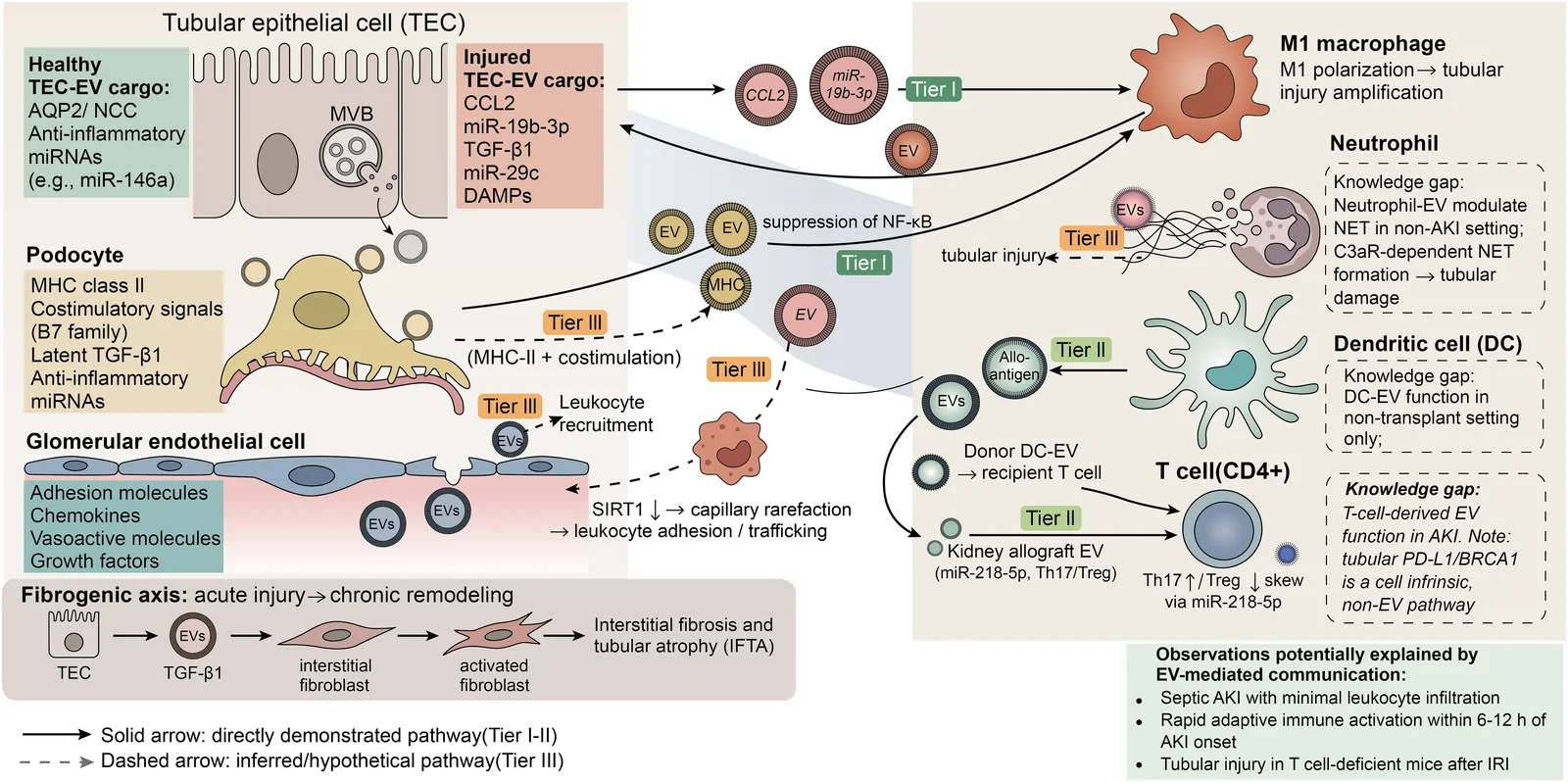
EV-mediated crosstalk between renal parenchymal cells and immune effectors in AKI. Solid arrows indicate pathways supported by direct experimental evidence (Tier I–II); dashed arrows (gray) indicate inferred or hypothetical pathways for which direct *in vivo* renal evidence is currently absent. Left – Renal parenchymal cells: Healthy tubular epithelial cells (TECs) release EVs containing AQP2 and NCC (70). Injured TECs release vesicles enriched in CCL2 (71), miR-19b-3p (4), TGF-β1 (73), and miR-29c (85). Podocytes constitutively release EVs with latent TGF-β1 and anti-inflammatory miRNAs that suppress NF-κB in resident macrophages (75); podocyte EVs also carry MHC class II and B7 family costimulatory signals for local T-cell activation (54, 79). Endothelial EVs transfer adhesion molecules and chemokines that shape leukocyte recruitment (81, 83). Right – Immune effectors: M1 macrophage–derived EVs amplify tubular injury and microvascular dysfunction (S-AKI) (89, 90). Neutrophil-derived EV and NETs are mobilized early via C3aR, amplifying complement-linked tubular injury (97) NET evidence is direct but neutrophil-EV evidence in kidney is limited. Donor DC-derived EVs traffic to recipient lymphoid organs and prime alloreactive T cells, with strongest evidence in the transplant context (99–101). Allograft EVs enriched in miR-218-5p skew the recipient Th17/Treg balance toward a pro-inflammatory phenotype (108). Fibrogenic axis (bottom): Injured TEC-derived TGF-β1–containing exosomes activate interstitial fibroblasts, driving interstitial fibrosis and tubular atrophy (73). Evidence-tier definitions: Tier I, direct *in vivo* AKI evidence (rodent or human) with functional or clinical validation; Tier II, kidney transplant or non-AKI kidney disease *in vivo* evidence; Tier III, *in vitro* renal cell/organoid evidence, or human biofluid evidence without paired renal functional validation. AQP2, aquaporin-2; NCC, Na^+^-Cl^-^ cotransporter; DC, dendritic cell; NET, neutrophil extracellular trap.

Similarly, direct evidence supporting a role for T-cell-derived EVs in AKI remains lacking. A recent report showed that PD-L1 promotes tubular repair after AKI through a T-cell-independent mechanism by interacting directly with BRCA1 to limit DNA damage (102). Although relevant, this finding pertains to cell-intrinsic parenchymal signaling rather than T-cell EV biology and should be distinguished from EV-associated interpretations of checkpoint pathways in the injured kidney. Collectively, EV-mediated communication between renal parenchymal cells and immune effectors constitutes an integrated relay network that addresses several paradoxes unexplained by classical leukocyte-centric models of AKI (**Figure 1**).

## 4 Etiology-stratified EV signaling in AKI

AKI is not a single disease but a syndrome, and EV cargo, cellular origin, and recipient tropism differ markedly across insult types (103–105). Throughout the etiology-specific discussion that follows, we distinguish endogenous EV mechanisms — signaling driven by EVs released from injured renal parenchymal or resident immune cells during the natural course of disease — from exogenous or engineered EV interventions, principally MSC-derived and other therapeutically administered EVs. This distinction is maintained explicitly within each subsection, since the therapeutic efficacy of administered EVs reflects the properties of the exogenous product rather than evidence for the corresponding endogenous signaling pathway in AKI. The four etiologies discussed below account for the majority of clinical AKI cases. This section traces how vesicular communication is reshaped at each disease stage, providing a framework for understanding why mechanistic insights derived from one subtype cannot be directly extrapolated to another (**Table 1**).

### 4.1 Allograft rejection–associated AKI

In kidney transplantation, alloimmune injury is the dominant driver of graft dysfunction (106). EV-mediated signaling in this context operates across at least three recognizable mechanisms: immune activation and allorecognition, diagnostic biomarker discovery, and maladaptive graft remodeling.

#### 4.1.1 Endogenous EV mechanisms

Vallabhajosyula et al. showed that donor tissue-specific EVs released into the recipient circulation traffic to the spleen and draining lymph nodes before clinical or histological signs of graft dysfunction appear (107), identifying them as early reporters of allograft status. Rutman et al. subsequently demonstrated that kidney allograft-derived EVs enriched in miR-218-5p skew the recipient Th17/Treg balance toward a pro-inflammatory phenotype, providing direct mechanistic evidence that donor EV cargo shapes alloreactive T-helper cell differentiation (108). Beyond acute immune activation, Franzin et al. showed that EVs derived from patients with antibody-mediated rejection induce tubular senescence and endothelial-to-mesenchymal transition in renal cells, establishing a mechanistic link between pathological allograft EVs and the cellular programs underlying chronic graft dysfunction (109).

#### 4.1.2 At the therapeutic interface

Wu et al. reported that MSC-derived EVs carrying lncRNA DANCR induce immune tolerance to kidney transplantation and attenuate rejection in a murine model, illustrating the immunomodulatory potential of exogenous EV-based interventions (110).

#### 4.1.3 Diagnostic and platform evidence

El Fekih et al. identified and validated a urinary exosome mRNA signature (CXCL9, CXCL11, CD3, CD4, IFNG, PRF1) capable of discriminating acute rejection from stable graft function and BK virus nephropathy, with performance comparable to donor-derived cell-free DNA (111). Park et al. further developed an integrated microfluidic platform for urinary transplant EV analysis, demonstrating the feasibility of non-invasive graft monitoring (112). Both represent proof-of-concept advances that have not yet entered routine clinical practice.

Together, the mechanistic, therapeutic, and diagnostic evidence reviewed above defines a multi-layered EV framework in kidney transplantation that spans early allorecognition, fibrogenic graft remodeling, and non-invasive monitoring — while each component awaits prospective, multicenter validation before clinical translation.

### 4.2 Sepsis-associated AKI

S-AKI arises within a systemic inflammatory milieu in which circulating EVs from activated leukocytes, endothelial cells, platelets, and visceral parenchymal cells contribute to the renal inflammatory milieu (113–115). Recent consensus reports have refined our understanding of S-AKI pathophysiology and underscored the need for etiology-stratified approaches (116).

#### 4.2.1 Endogenous pathogenic EV mechanisms

The pathogenic EV landscape in S-AKI involves multiple cellular sources and converging mechanisms. During the early bacteremic phase, plasma EVs from patients with septic shock carry inflammatory cytokines, NF-κB- and cell-cycle-related microRNAs, and reactive oxygen species generators — evidence from human plasma, though with renal effects that remain to be directly demonstrated in renal tissue (117, 118). At the peak of renal inflammation, septic macrophage-derived EVs target glomerular endothelial cells and suppress SIRT1, contributing to capillary rarefaction and impaired oxygen delivery in rodent S-AKI models supported by matched *in vitro* studies (89). Platelet-derived EVs have been shown to aggravate septic AKI by delivering ADP-ribosylation factor 6 (ARF6) to tubular cells, amplifying endosomal trafficking and injury propagation (119). More recently, TEC-derived EVs carrying serum amyloid A1 (SAA1) were shown to exacerbate S-AKI by promoting NET formation in a paracrine manner, illustrating a TEC-to-neutrophil vesicular axis that links tubular stress to innate immune amplification (120).

#### 4.2.2 Endogenous reparative EV mechanisms

Not all EVs in S-AKI propagate injury. Endothelial progenitor cell-derived EVs containing miR-93-5p confer protection against sepsis-induced AKI through suppression of the KDM6B/H3K27me3/TNF-α axis (121), and a related study demonstrated that endothelial progenitor cell-derived EVs carrying miR-21-5p attenuate S-AKI by inhibiting RUNX1 expression (122). Pan et al. further reported that remote ischemic preconditioning generates circulating EVs enriched in miR-21, which suppress programmed cell death and reduce S-AKI severity in rodent models (123). Together, these findings suggest that the septic EV milieu is shaped by competing pathogenic and reparative signals, and that conditioning interventions may shift this balance toward protection.

#### 4.2.3 Biomarker evidence

Li et al. applied single-vesicle urinary EV proteomics to patients with S-AKI and identified complement receptor CD35 (CR1) as a biomarker distinguishing S-AKI from non-septic AKI and from sepsis without renal involvement, combining human cohort validation with single-vesicle proteomic resolution (124). This study illustrates how the evolving single-vesicle analytical toolkit described in Section 6 can be deployed to address the clinical diagnostic challenge of S-AKI phenotyping.

#### 4.2.4 Therapeutic interface

Urine-derived stem cell exosomes have been shown to attenuate sepsis-related AKI in experimental models by suppressing tubular inflammation and oxidative stress (125), adding to the growing body of evidence supporting exogenous EV-based interventions in S-AKI. It should be noted that these therapeutic studies reflect the properties of administered exogenous EVs rather than endogenous injury signals, a distinction emphasized throughout Section 4.

Collectively, the S-AKI EV literature spans a spectrum of evidence maturity, from validated human biomarker studies (124) to mechanistic rodent experiments (89, 120–122) and early-stage therapeutic models (123, 125). The emerging picture is one of substantial EV diversity across cell type, phase, and function — a complexity that demands etiology-stratified, stage-resolved investigation rather than a single unifying mechanistic model.

### 4.3 Nephrotoxic AKI

Nephrotoxic AKI, exemplified by cisplatin chemotherapy and iodinated contrast media, is characterized by relatively cell-autonomous tubular injury with modest early leukocyte infiltration (126). Early proteomic work by Zhou et al., combining a rat cisplatin nephrotoxicity model with urinary specimens from ICU patients with AKI, identified fetuin-A as an EV-associated urinary marker whose appearance tracks tubular injury, providing patient-level evidence that nephrotoxic insults reshape the urinary EV cargo landscape (86). In a rodent cisplatin AKI model, Xu et al. subsequently demonstrated that transcription factor EB (TFEB), a master regulator of lysosomal and autophagosomal biogenesis, protects against cisplatin-induced injury by regulating the exosome-multivesicular body (MVB) pathway, thereby attenuating tubular apoptosis and limiting the release of pro-inflammatory EV cargo (127). Beyond these two direct renal studies, primary evidence dissecting how endogenous renal parenchymal EVs orchestrate immune remodeling in drug- or toxin-induced AKI remains sparse. Most current mechanistic proposals in this setting draw on cross-organ analogy — chiefly hepatocyte-derived EV microRNAs reshaping Kupffer cell and monocyte phenotypes in acetaminophen liver injury and epithelial DAMP-EVs activating pulmonary myeloid cells in bleomycin lung injury (128, 129). Interpreted strictly as analogy rather than as renal evidence, these observations motivate the hypothesis that stressed tubular cells release EVs conveying toxic-stress signals to intrarenal mononuclear phagocytes. However, the existence, dominant cargo, and recipient-cell specificity of such circuits must be validated in nephrotoxic AKI models before this hypothesis can be considered established.

### 4.4 Ischemia-reperfusion injury–associated AKI

IRI-AKI is the most extensively studied AKI model and a frequent complication of both cardiovascular surgery and renal transplantation (130). Contrary to the impression given by some earlier reviews (131), a growing body of evidence now characterizes endogenous EV-mediated communication between tubular epithelial cells, endothelial cells, macrophages, and other immune effectors following renal IRI, though several mechanistic links remain to be defined.

#### 4.4.1 Endogenous pathogenic EV mechanisms

TEC-derived EVs are established contributors to the pathological response in renal IRI across multiple cargo axes. Wang et al. demonstrated that tubular cell-derived EVs carrying miR-491-3p aggravate IRI by inhibiting macrophage SIRT1-mediated Notch intracellular domain deacetylation, thereby preventing ubiquitin-proteasome degradation of inflammatory mediators and sustaining M1 macrophage activation (132). Integrin β1-enriched EVs released by injured kidney cells recruit a specific Fn1+ macrophage subset to the site of injury, amplifying ischemia-reperfusion-induced inflammation through integrin-mediated macrophage homing (133). Li et al. showed that exosome-delivered inhibition of miR-106b-5p mitigates AKI by reducing transmissible endoplasmic reticulum stress and restraining M1 macrophage polarization (134), while Yuan et al. demonstrated that macrophage-derived exosomal miR-195a-5p impairs mitochondrial function in TECs, establishing a bidirectional macrophage-tubule EV axis in IRI-AKI (135). Under hypoxic conditions, tubular exosomes enriched in miR-20a-5p have been shown to modulate tubular injury responses through an autocrine-paracrine mechanism (136). Chen et al. further identified a circadian clock-dependent axis in which BMAL1 regulates TEC-derived exosomal miR-27a-3p to suppress macrophage-myofibroblast transition and alleviate IRI-induced renal fibrosis (137). Together, these studies document a mechanistically heterogeneous but coherent endogenous EV network connecting tubular stress, macrophage reprogramming, and fibrogenic transition in renal IRI.

#### 4.4.2 Complement-neutrophil EV axis

The innate effector response in IRI involves both neutrophil-derived EVs and NETs, which are distinct but potentially interacting processes as discussed in Section 3.3. Han et al. demonstrated that genetic deletion of C3aR reduces NET formation and tubular damage in murine renal IRI (97), providing direct *in vivo* evidence for the complement-neutrophil axis while not directly addressing EV-specific mechanisms. This axis is referenced here for completeness; its mechanistic interpretation is developed in Section 3.3.

#### 4.4.3 Fibrogenic transition

Borges et al. established that TGF-β1-containing exosomes from injured TECs activate interstitial fibroblasts and initiate the regenerative-to-fibrogenic switch (73), a finding extended by the observation that senescent TEC-derived EVs transport miR-20a and miR-21 to macrophages and induce macrophage-to-myofibroblast transition during the fibrotic phase of IRI (74).

#### 4.4.4 Therapeutic MSC-EV evidence (exogenous EVs)

A systematic review and meta-analysis documented consistent renoprotective effects of exogenous MSC-EVs across preclinical IRI models (138). MSC-EVs attenuate IRI via multiple cargo-specific mechanisms, including delivery of miR-100-5p targeting the FKBP5/AKT axis (139), stabilization of mitochondrial DNA to reduce mitochondrial damage and inflammation (140), targeting of the CH25H/25HC-ferroptosis axis (141), and delivery of miR-202-5p via umbilical cord MSC-EVs targeting the GOLIM4/PI3K/AKT pathway (142). It should be emphasized that these therapeutic effects reflect the properties of exogenously administered MSC-derived EVs rather than endogenous injury signals; mechanistic insights from this literature should not be extrapolated to characterize the behavior of endogenous renal EVs in IRI.

The IRI-AKI EV field has advanced substantially in recent years, with direct renal evidence now available for multiple TEC-macrophage cargo axes. Defining the upstream triggers for cargo loading, the cell-of-origin specificity of these EVs *in vivo*, and their interactions with the complement-neutrophil and endothelial compartments remains the central challenge for the next phase of investigation.

## 5 Stage-specific roles: initiation, amplification, resolution, and maladaptive repair

The four etiologies described above share a common temporal architecture in which EV cargo and function evolve across disease phases. The framework presented below is a synthesizing hypothesis that integrates available cross-etiology evidence; specific stage assignments should be regarded as model-derived rather than as established disease trajectories. Several boundaries between stages are expected to overlap with experimental data once they become available, and the framework should be tested through prospective time-resolved EV profiling in human AKI cohorts.

### 5.1 Initiation

In the earliest phase, parenchymal EVs enriched in DAMPs and complement fragments act as sterile danger signals that translate cellular stress into immune activation before massive leukocyte infiltration. However, immune activation and epithelial stress responses overlap temporally in many AKI models, and the boundary drawn here is therefore approximate rather than absolute. This pattern has been documented in rodent and *in vitro* tubular injury studies (71, 85).

### 5.2 Amplification

As inflammation peaks, immune cell-derived EVs (particularly those from classically activated macrophages in rodent sepsis-AKI models (89, 90) and from complement-linked neutrophils and NETs in renal IRI (97))target TECs, glomerular endothelial cells, and peritubular endothelial cells, propagating tubular injury and microvascular dysfunction. Septic macrophage-derived exosomes additionally impair endothelial SIRT1 signaling, as demonstrated in a rodent S-AKI model supported by matching *in vitro* endothelial studies (89).

### 5.3 Resolution

During the recovery phase, certain EV populations acquire protective properties. For example, miR-21-enriched exosomes generated after remote ischemic preconditioning suppress programmed cell death and attenuate S-AKI in rodent studies (123). Whether MSC-EVs or other therapeutically engineered vesicles engage endogenous resolution circuits or act through distinct mechanisms remains to be determined.

### 5.4 Maladaptive repair

During the transition to chronic injury, TEC-derived TGF-β1-containing exosomes activate interstitial fibroblasts and drive the regenerative-to-fibrogenic switch, as shown directly in rodent renal IRI (73). Recent integrative spatial and single-cell analyses have identified a distinct ECM-remodeling macrophage subset that emerges during the AKI-to-CKD transition, suggesting that EV-producing macrophage identities are themselves remodeled across disease stages and contribute to maladaptive repair (143). Single-cell RNA sequencing has revealed both homogeneity and heterogeneity in cytopathological mechanisms across different AKI etiologies (144), while integration of single-cell and spatial transcriptomics has uncovered neutrophil diversity and spatial heterogeneity within the injured kidney (145). These high-resolution profiling approaches now allow EV-producing macrophage subsets to be mapped to discrete disease stages with cellular precision. In the transplant setting, Quaglia et al. reviewed evidence suggesting that persistent release of fibrogenic and senescence-associated EV cargo contributes to interstitial fibrosis and tubular atrophy (87); however, as discussed in Section 4.1, this remains a hypothesis awaiting prospective, multicenter, etiology-stratified validation. Stage-specific EV signatures therefore offer a candidate framework for monitoring the trajectory from acute injury toward chronic dysfunction, pending validation in prospective cohorts.

## 6 Technologies for resolving EV heterogeneity in AKI research

**Figure 2:**
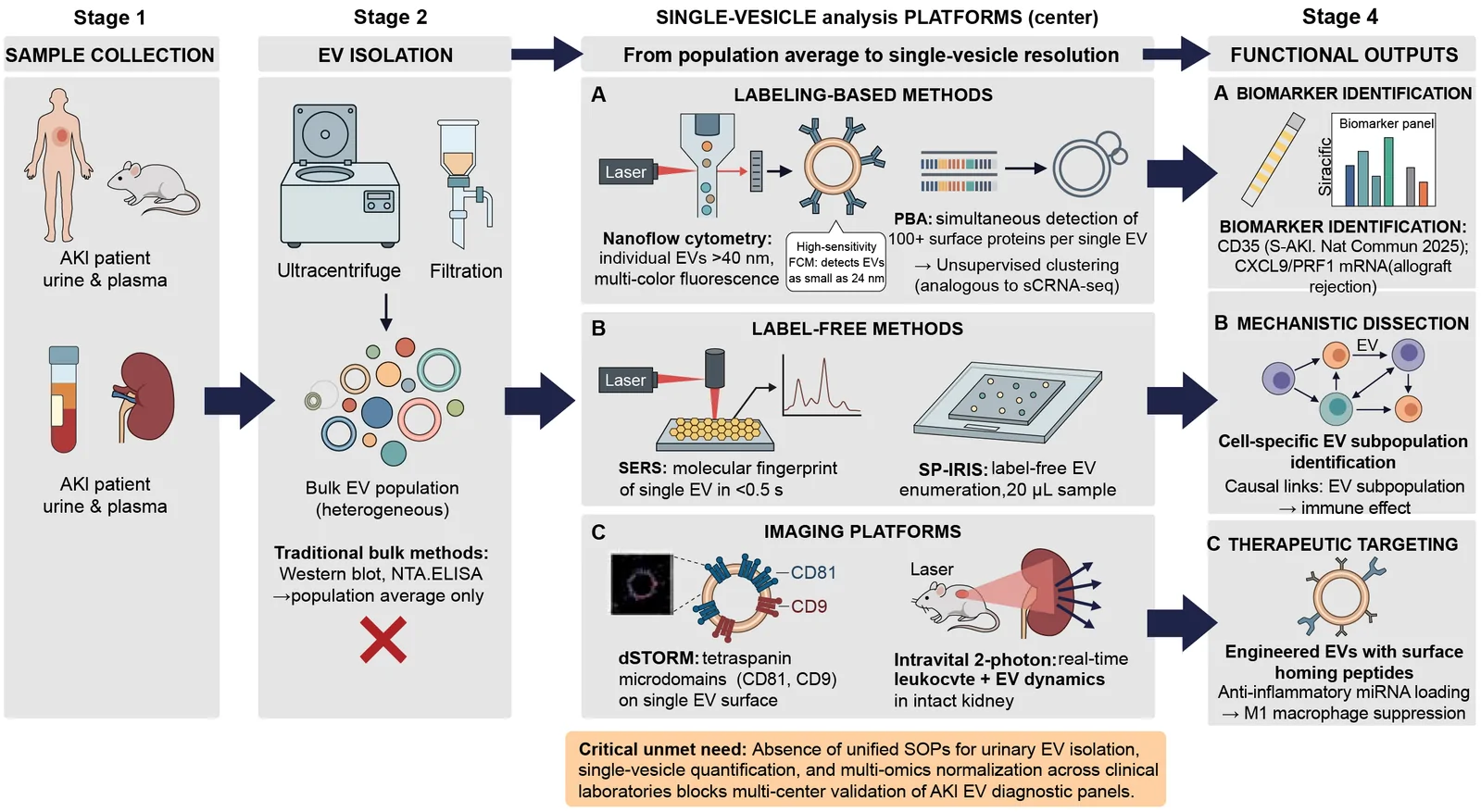
Single-vesicle analytical platforms for resolving EV heterogeneity in AKI. Workflow from bulk EV isolation to single-vesicle resolution and clinical translation. Stage 1 – Sample collection: Urine and plasma from AKI patients and preclinical models are processed for EV enrichment. Platform-dependent increases in urinary and plasma EV abundance have been reported in early-phase injury studies; the magnitude of change is highly dependent on isolation method, calibration standard, and patient subset, and quantitative thresholds should therefore not be generalized across platforms. Stage 2 – EV isolation: Ultracentrifugation and filtration yield heterogeneous EV populations; traditional bulk readouts (Western blot, NTA, ELISA) report only population averages and obscure functional heterogeneity. Stage 3 – Single-vesicle analysis platforms: **(A)** Labeling-based methods, including nano-flow cytometry (individual EVs >~40 nm) (150), high-sensitivity flow cytometry (down to ~24 nm) (151, 152), droplet digital ExoELISA (153), multiplex bead-based flow cytometry (149), and proximity barcoding assay (PBA) generating digital single-vesicle proteomic profiles analogous to scRNA-seq (147, 148). **(B)** Label-free methods: Surface-enhanced Raman spectroscopy (SERS) uses gold or silver nanoparticle substrates to substantially enhance signal sensitivity, enabling rapid acquisition of clear Raman spectra from individual EVs within approximately 0.5 seconds under specific instrumentation and substrate conditions (156). Single-particle interferometric reflectance imaging sensing (SP-IRIS) enables label-free capture and enumeration of individual EVs from as little as 20 μL of purified sample, with platform- and protocol-dependent sensitivity (157). **(C)** Imaging platforms, including direct stochastic optical reconstruction microscopy (dSTORM) that resolves tetraspanin (CD81, CD9) microdomains on individual EVs (168), and intravital two-photon microscopy for real-time leukocyte and EV dynamics in the intact kidney (167). Stage 4 – Functional outputs: **(A)** Etiology-stratified biomarker identification, exemplified by urinary CD35 in S-AKI (124) and the CXCL9/CXCL11/CD3/CD4/IFNG/PRF1 mRNA panel in allograft rejection (111). **(B)** Mechanistic dissection of cell-specific EV subpopulations and their causal links to immune effects. **(C)** Therapeutic targeting via engineered EVs carrying homing peptides and anti-inflammatory miRNA cargo. FCM, flow cytometry; NTA, nanoparticle tracking analysis; PBA, proximity barcoding assay; SERS, surface-enhanced Raman spectroscopy; SP-IRIS, single-particle interferometric reflectance imaging sensing; dSTORM, direct stochastic optical reconstruction microscopy; S-AKI, sepsis-associated acute kidney injury.

Understanding the role of EVs in renal immune regulation has depended heavily on advances in analytical technologies. Conventional EV studies have relied largely on bulk population-level analyses that reflect average vesicle properties and obscure functional heterogeneity. The platforms reviewed below are organized according to a practical distinction: those that have already been deployed in renal EV research and have generated AKI-relevant findings, and those that represent emerging capabilities with clear but as yet unrealized potential for the field (146). The organization of these categories is illustrated in **Figure 2**.

### 6.1 Single-vesicle proteomics and phenotyping - applied to renal EV biology

Several single-vesicle platforms have already been applied to clinically meaningful questions in AKI. Proximity barcoding assays generate digital single-vesicle proteomic profiles analogous to single-cell RNA sequencing (147, 148) and are capable of resolving cell-of-origin ambiguity in mixed urinary EV populations — a key limitation of bulk urinary EV analyses highlighted in Section 7.3. The clinical utility of such resolution was demonstrated by Li et al., who used single urinary EV proteomics to identify complement receptor CD35 as a biomarker distinguishing S-AKI from non-septic AKI (124). Multiplex bead-based flow cytometry has been applied to discriminate donor- from recipient-derived EVs in kidney transplantation (149), directly addressing the cell-of-origin problem in the transplant setting. High-sensitivity nano-flow cytometry detects individual EVs larger than approximately 40 nm and supports multiparameter phenotyping (150–152), while droplet digital ExoELISA enables absolute EV counting at high sensitivity (153). These platforms collectively underpin the etiology-stratified biomarker signatures reviewed in Section 4, including the urinary CD35 signature (124) and the CXCL9/CXCL11/CD3/CD4/IFNG/PRF1 mRNA panel (111). Kim-1-targeted EVs have been developed as a delivery platform that selectively homes to injured proximal tubules, exploiting the disease-induced surface marker landscape to direct therapeutic cargo (154); this work also illustrates how single-vesicle surface profiling informs targeted delivery design.

### 6.2 Label-free methods — established platforms with emerging renal applications

Label-free techniques offer molecular fingerprinting without antibody labeling and are well established in general EV characterization, though their direct application to renal AKI remains limited and represents an area of growth. Raman spectroscopy provides molecular fingerprint information from individual EVs without labeling (155). Surface-enhanced Raman spectroscopy (SERS), using gold or silver nanoparticle substrates, substantially enhances signal sensitivity and has been shown to acquire clear Raman spectra from individual EVs within approximately 0.5 seconds under optimized substrate conditions (156). Single-particle interferometric reflectance imaging sensing (SP-IRIS) enables label-free capture and enumeration of individual EVs from as little as 20 μL of purified sample (157). These label-free platforms have not yet been directly applied to renal EV cohorts in published AKI studies, but their compatibility with unlabeled biofluid samples makes them well suited to future urinary and plasma EV profiling in this setting. Newer approaches — including rapid optical trapping of nanoscale vesicles in solution (158), multiplexed platforms integrating surface protein detection with cargo analysis (159, 160), single-molecule confocal fluorescence microscopy (161), double digital assays (162), and rolling circle amplification-expansion microscopy (163) — remain, at present, general-purpose EV characterization tools awaiting dedicated deployment in renal disease cohorts.

### 6.3 Intravital and *in vivo* tracking — applied to renal EV biodistribution

This category includes technologies with direct precedent in kidney research. Near-infrared fluorescence and bioluminescent reporter systems have been used to track EV biodistribution *in vivo* (164, 165), and Grange et al. demonstrated that MSC-derived EVs preferentially accumulate in injured kidneys and are retained within the renal interstitium in an experimental AKI model (166) — direct evidence that intravital imaging can resolve renal-specific EV trafficking. Intravital two-photon microscopy has been used to resolve leukocyte behavior in glomerular and tubulointerstitial compartments at depths of approximately 100 μm (167); while this technique has been validated for immune-cell dynamics in the kidney, its extension to real-time EV tracking within the same anatomical compartments remains an emerging application. Superresolution approaches such as direct stochastic optical reconstruction microscopy (dSTORM) have demonstrated that tetraspanin proteins including CD81 and CD9 are organized into discrete surface microdomains rather than being uniformly distributed on individual EVs (168); this spatial resolution has not yet been applied to renal-derived EVs specifically but offers a plausible route to mapping immune-recognition motifs on kidney-derived vesicles.

### 6.4 Kidney organoid and organ-on-chip systems— an emerging platform for AKI-specific EV research

Kidney organoid technology has advanced rapidly and now recapitulates key features of AKI pathophysiology, providing a three-dimensional, multicellular context in which EV-immune interactions can be investigated under controlled conditions (169–171). Organoids combined with immune cell coculture preserve spatial relationships among TECs, podocytes, endothelial cells, and interstitial components (172–174), offering a platform for functional dissection of EV-immune crosstalk that is not achievable in two-dimensional culture (175). Organ-on-chip platforms extend this capability by introducing fluid shear stress and vascular perfusion (176, 177). It has been hypothesized, though not yet experimentally validated, that the hemodynamic forces reproduced in these systems could influence TEC gene expression, cell polarity, and, by extension, the secretory phenotype and EV cargo composition of renal epithelial and endothelial cells (178). This hypothesis — hemodynamic regulation of EV cargo — awaits dedicated experimental testing in AKI-relevant organ-on-chip or *in vivo* models before it can be considered an established mechanism. At present, kidney organoid and organ-on-chip systems represent the platform class with the greatest gap between technical maturity and AKI-specific EV application, and closing this gap is a priority for the next phase of methodological development in the field.

## 7 Translational barriers and future directions

### 7.1 Sample-source variability

Urinary and plasma EVs differ in cellular origin, dynamic range, and susceptibility to pre-analytical variables such as diurnal fluctuation, hydration status, and freeze-thaw cycles. Cross-cohort comparisons remain difficult without MISEV2023-compliant collection SOPs, and few studies have directly compared urinary and plasma EV performance within the same AKI cohort.

### 7.2 Isolation and detection standardization

Ultracentrifugation, size-exclusion chromatography, tangential flow filtration, and immunoaffinity capture yield partially non-overlapping vesicle populations; apparent mechanistic differences between studies may therefore reflect isolation bias. Single-vesicle detection platforms remain confined to specialized laboratories, and their translation into routine ICU or transplant workflows is limited by throughput, cost, and operator dependence.

### 7.3 Cargo attribution and cell-of-origin uncertainty

Bulk urinary or plasma analyses cannot resolve which renal or extrarenal cell released a given vesicle, and overlapping surface markers complicate the distinction between parenchymal and immune sources. This limitation hinders etiology-stratified biomarker development but can be mitigated through cell-specific surface panels, PBA-based proteomic clustering, and organ-on-chip source mapping.

### 7.4 Therapeutic EV batch variability and delivery safety

MSC-EV and engineered EV preparations exhibit batch-to-batch variability in cargo, potency, and biodistribution. Standardization, bioengineering, and clinical translation of MSC-derived EVs in regenerative medicine remain active areas of development (179). Pleiotropic mechanisms of MSC-EV action have been documented in chronic kidney disease (180), further motivating the need for rigorous manufacturing and quality control protocols. This issue has been systematically addressed in a recent head-to-head comparison of MSC-EVs produced under fetal bovine serum versus human platelet lysate culture conditions, which demonstrated substantial divergence in EV cargo and functional profile attributable to manufacturing input rather than donor cell source (181). Dose-response relationships, repeat-dosing safety, and long-term renal biodistribution remain incompletely characterized, and regulatory pathways for these products in critically ill AKI populations are not yet defined. A broader class of EV-based nanotherapeutics, including those encapsulating anti-inflammatory cytokines, miRNAs, or small-molecule drugs, has recently entered preclinical evaluation for renal inflammatory disease (182).

### 7.5 Cohort-based clinical validation

Existing EV biomarker studies in AKI, though internally validated, have not been reproduced in prospective, multicenter, etiology-stratified cohorts. A 2024 review of biomarkers in AKI underscored the translational gap between discovery and clinical implementation (183), emphasizing the need for prospective, multicenter validation and integration with existing diagnostic frameworks such as KDIGO criteria. Biomarker adoption is further complicated by variable AKI definitions (KDIGO stage, timing of onset) and low pretest probability in mixed ICU populations. Etiology-stratified prospective registries that integrate urinary and plasma single-vesicle analysis with donor-derived cell-free DNA and clinical KDIGO criteria represent a plausible next step.

### 7.6 Priority research gaps

Several specific gaps recur throughout this review and warrant explicit prioritization. Broadly, outstanding challenges span methodological standardization, translational validation, and unresolved mechanistic questions. From a methodological and translational perspective, future studies need to address five core priorities: first, the *in vivo* identification of EV-originating cell populations via lineage-tracing and proximity-barcoding technologies to define cellular sources of pathogenic and protective EVs in the injured kidney; second, rigorous functional validation of disease-associated EV cargo, which requires conditional knockdown of cargo-loading machinery in EV-producing cells to verify causal bioactivity; third, the establishment of definitive relationships between altered EV signatures and progressive AKI trajectories, achievable through longitudinal clinical cohort analyses integrated with standardized KDIGO staging systems; fourth, standardized reporting of pre-analytical variables across EV studies, including urine concentration, freeze–thaw cycles, storage duration, and patient hydration status, to reduce technical variability and improve reproducibility; fifth, the combinatorial integration of EV molecular profiles with established clinical indices—such as KDIGO stage, AKI etiology, and disease severity scores—to refine patient AKI phenotyping. Beyond these methodological and translational bottlenecks, three prominent mechanistic gaps require dedicated investigation to clarify EV-mediated immune regulation in AKI: (i) the mechanistic role of DC-derived EVs in non-transplant AKI, currently supported only by transplant models; (ii) the role of T-cell–derived EVs in AKI, for which no direct *in vivo* evidence exists and which should be investigated independently of cell-intrinsic checkpoint phenomena such as tubular PD-L1/BRCA1 signaling (87); and (iii) endogenous EV-mediated immune signaling in renal ischemia-reperfusion injury.

## 8 Concluding perspective

The evidence in this review supports reframing AKI as a syndrome in which dysregulated vesicular communication between renal parenchymal cells and immune effectors shapes disease trajectory beyond intrinsic tubular injury. With single-vesicle analytics, kidney organoids, and etiology-stratified cohorts now in hand, the field can move beyond describing EV cargo toward defining which vesicular circuits drive specific AKI subtypes and disease stages, and whether they can be modulated therapeutically. Three translational priorities emerge from this synthesis: engineered EVs as immunomodulatory agents, validation of stage-specific EV signatures in prospective multicenter cohorts, and integration of EV-based readouts with clinical criteria to refine AKI phenotyping. Achieving these milestones would represent an important step toward shifting AKI management from supportive care toward interventions matched to the immunological state of the injured kidney. While EV-based biomarkers and therapeutics hold promise to advance personalized strategies for AKI management, substantial preclinical and prospective clinical validation across diverse patient cohorts is mandatory before these approaches can be reliably integrated into routine clinical care.

## Supplementary material

Supplementary Table 1 Cross-Organ Analogies Informing EV-Mediated Immune Signaling in AKI (Tier IV Evidence).
