# Supplementary material for: Extracellular vesicles mediate immune regulation in acute kidney injury
Source: Front Immunol. 2026 Sep 1;17:1952566. doi: 10.3389/fimmu.2026.1952566 (PMC13575969; doi:10.3389/fimmu.2026.1952566)
Supplement: Supplementary file 1 [file Table1.docx]

**Supplementary Table S1 | Cross-Organ Analogies Informing EV-Mediated Immune Signaling in AKI (Tier IV Evidence)**

| Injury context | EV source (cell of origin) | Representative cargo | Recipient cell/target | Immune/functional effect | Evidence tier | Evidence type | Key reference |
| --- | --- | --- | --- | --- | --- | --- | --- |
| Cross-organ analogy: hepatotoxic injury | Hepatocyte-derived EVs | EV microRNAs | Kupffer cells, monocytes | Inflammatory reprogramming in acetaminophen liver injury | IV — Liver injury; no direct renal in vivo evidence | Mechanistic (non-renal) | Hirsova 2016 **(128)** |
| Cross-organ analogy: pulmonary injury | Epithelial cell–derived microvesicles | DAMP-enriched cargo | Pulmonary macrophages, dendritic cells | Myeloid activation in bleomycin lung injury | IV — Lung injury; no direct renal in vivo evidence | Mechanistic (non-renal) | Lee 2016 **(129)** |

This table lists Tier IV cross-organ analogy entries removed from the main Table 1. These studies provide mechanistic evidence in non-renal injury settings and are retained here to support hypothesis generation regarding EV-mediated immune signaling in nephrotoxic AKI and IRI-AKI, where direct renal evidence remains limited. They should not be interpreted as direct evidence for EV-mediated immune regulation in the kidney. Evidence-tier definitions follow those in Table 1.
